# Cord Blood CD8^+^ T Cells Have a Natural Propensity to Express IL-4 in a Fatty Acid Metabolism and Caspase Activation-Dependent Manner

**DOI:** 10.3389/fimmu.2018.00879

**Published:** 2018-04-25

**Authors:** Yuxia Zhang, Jovana Maksimovic, Bing Huang, David Peter De Souza, Gaetano Naselli, Huan Chen, Li Zhang, Kai Weng, Hanquan Liang, Yanhui Xu, John M. Wentworth, Nicholas D. Huntington, Alicia Oshlack, Sitang Gong, Axel Kallies, Peter Vuillermin, Min Yang, Leonard C. Harrison

**Affiliations:** ^1^Guangzhou Institute of Pediatrics, Guangzhou Women and Children’s Medical Center, Guangzhou Medical University, Guangzhou, China; ^2^Walter and Eliza Hall Institute of Medical Research, Parkville, VIC, Australia; ^3^Department of Medical Biology, University of Melbourne, Parkville, VIC, Australia; ^4^Murdoch Children’s Research Institute, Royal Children’s Hospital, Parkville, VIC, Australia; ^5^Department of Pediatrics, University of Melbourne, Parkville, VIC, Australia; ^6^Bio21 Institute, University of Melbourne, Parkville, VIC, Australia; ^7^School of Data and Computer Science, Sun Yat-sen University, Guangzhou, China; ^8^Barwon Health, Geelong, VIC, Australia; ^9^Deakin University, Geelong, VIC, Australia

**Keywords:** CD8^+^ T cell, glycolysis, fatty acid oxidation, caspases, IL-4

## Abstract

How T cells differentiate in the neonate may critically determine the ability of the infant to cope with infections, respond to vaccines and avert allergies. Previously, we found that naïve cord blood CD4^+^ T cells differentiated toward an IL-4-expressing phenotype when activated in the presence of TGF-β and monocyte-derived inflammatory cytokines, the latter are more highly secreted by infants who developed food allergy. Here, we show that in the absence of IL-2 or IL-12, naïve cord blood CD8^+^ T cells have a natural propensity to differentiate into IL-4-producing non-classic T_C_2 cells when they are activated alone, or in the presence of TGF-β and/or inflammatory cytokines. Mechanistically, non-classic T_C_2 development is associated with decreased expression of IL-2 receptor alpha (CD25) and glycolysis, and increased fatty acid metabolism and caspase-dependent cell death. Consequently, the short chain fatty acid, sodium propionate (NaPo), enhanced IL-4 expression, but exogenous IL-2 or pan-caspase inhibition prevented IL-4 expression. In children with endoscopically and histologically confirmed non-inflammatory bowel disease and non-infectious pediatric idiopathic colitis, the presence of TGF-β, NaPo, and IL-1β or TNF-α promoted T_C_2 differentiation *in vitro*. *In vivo*, colonic mucosa of children with colitis had significantly increased expression of IL-4 in CD8^+^ T cells compared with controls. In addition, activated caspase-3 and IL-4 were co-expressed in CD8^+^ T cells in the colonic mucosa of children with colitis. Thus, in the context of colonic inflammation and limited IL-2 signaling, CD8^+^ T cells differentiate into non-classic T_C_2 that may contribute to the pathology of inflammatory/allergic diseases in children.

## Summary

Inflammation limits IL-2 signaling and promotes differentiation of cord blood CD8^+^ T cells into an IL-4^+^ phenotype, requiring fatty acid metabolism and caspase activation. Such cells co-expressing activated caspase-3 and IL-4 can be identified in the mucosa of children with colitis.

## Introduction

Differentiation of naïve CD4^+^ T cells into functionally distinct helper T cell (T_H_) lineages such as T_H_1 (IFN-γ), T_H_2 (IL-4), T_H_17 (IL-17), and T_FH_ (IL-21) cells and induced regulatory T cells (iTreg) that express FOXP3 is shaped by the cytokine environment (1). This reflects the nature of local tissues (e.g., mucosal or systemic lymphoid), as well as innate immune inflammatory responses to pathogens and other stimuli (2). Similarly, naïve CD8^+^ T cells differentiate into T_C_1 (IFN-γ) and T_C_2 (IL-4) (3), T_C_17 (IL-17) (4), T_FC_ (5), or FOXP3-expressing (T_CREG_) (6) cells in the presence of the appropriate cytokines.

Human neonates display differences in innate and adaptive immune cell phenotypes and functions compared with adults. Cord blood mononuclear cells stimulated with toll-like receptor agonists secrete less IFN-α and IL-12 but more IL-6, a cytokine shift that inhibits T_H_1 differentiation and may contribute to increased neonatal susceptibility to infection by intracellular pathogens and viruses (7). The intrinsic differentiation capacity of cord blood CD8^+^ T cells is not impaired but differs from adults, with increased propensity to proliferation and cell death (8). Differentiation of neonatal CD8^+^ T cells under inflammatory conditions is not well understood but may be relevant to the infant’s response to infections and vaccines and susceptibility to diseases such as allergies. Increased inflammatory cytokine (IL-1β, IL-6, and TNF-α) production in cord blood in response to innate stimuli (9–11) and persistence of type 2/IL-4 immunity in the first year of life (12) have been associated with allergic sensitization. Previously, we reported that TGF-β and inflammatory cytokines, the latter secreted in higher amounts by cord blood monocytes from infants who developed food allergy, suppressed IL-2 expression by human cord blood CD4^+^ T cells and promoted a non-classical IL-4 T_H_2-type phenotype in naïve CD4^+^ T cells and naïve natural regulatory CD4^+^ T cells (13). This prompted us to investigate the differentiation of naïve cord blood CD8^+^ T cells in the presence of inflammatory cytokines. We found that in the absence of IL-2 or IL-12, naïve cord blood CD8^+^ T cells have a natural propensity of differentiating into IL-4-producing non-classic T_C_2 cells, associated with decreased glycolysis and increased fatty acid metabolism and caspase-dependent cell death. Similar cells were identified *in situ* in the colon of children with endoscopically and histologically confirmed non-inflammatory bowel disease (IBD) and non-infectious pediatric idiopathic colitis (PIC).

## Materials and Methods

### Subjects

This study was carried out in accordance with the recommendations of the International Ethical Guidelines for Research Involving Human Subjects. The protocols were approved by the Human Ethics Committees of Walter and Eliza Hall Institute, Barwon Health, Geelong, and Guangzhou Women and Children’s Medical Center (GWCMC). Legal guardians of all subjects gave written informed consent in accordance with the Declaration of Helsinki. Cord blood and colon biopsies were obtained from Barwon Infant Study (14) and hospitalized children at GWCMC (Ethics Number 2017072601). Children (*n* = 18) at GWCMC underwent colonoscopy and biopsy for the diagnostic evaluation of chronic abdominal pain, vomiting, or lower gastrointestinal bleeding (Table S3 in Supplementary Material). Colitis was identified endoscopically as having mucosal swelling, hyperemia, erosion, or ulceration and histologically as impaired epithelial integrity, increased lymphocytes and/or eosinophil [>20/HPF (high power field)] infiltrations. Control colonic biopsies were from children who displayed no features of colitis on colonoscopy and histology.

### CD8^+^ T Cell Isolation and Activation

Frozen cord blood mononuclear cells were thawed and surface stained with anti-TCRαβ, -CD4, -CD8a, -CD45RA, -CD25, -CD14, -CD16, and HLA-DR antibodies. Naïve CD8^+^ T cells (CD14^−^CD16^−^TCRαβ^+^CD4^−^CD8a^+^CD45RA^+^CD25^−^) were sorted by flow cytometry. Naïve CD8^+^ cells were activated with antihuman CD3/CD28 antibody microbeads (Life Technologies) at 1:1 ratio in the presence of recombinant human cytokines in IP5 medium [Iscove’s modified Dulbecco’s medium (Life Technologies) supplemented with 5% pooled human serum, 2 mM glutamine, 0.05 mM 2-mercaptoethanol, 100 U/ml penicillin, 100 µg/ml streptomycin, and 100 µM non-essential amino acids]. On day 3, cultures were supplemented with cytokine-containing fresh IP5 medium. The final concentrations of cytokines used were as follows: IL-2 (200 U/ml), IL-4 (10 ng/ml), IFN-γ (10 ng/ml), IL-1β (10 ng/ml), TNF-α (10 ng/ml), and IL-6 (100 ng/ml). TGF-β was used at 5 ng/ml for IL-2 + TGF-β and at 1 ng/ml elsewhere. Short chain fatty acids were supplemented at 1 mM. Pan-caspase inhibitor QVD-Oph (50 µM) was added in some experiments at day 1 or day 3. On days 4–5, activated T cells were re-stimulated with PMA (100 ng/ml) and ionomycin (1 µM) in the presence of monensin (2 µM) for 5 h and stained intracellularly with anti-pS6, -T-bet, -GATA-3, -IFN-γ, -IL-4, and -IL-2 antibodies. Activated T cells were also surface stained with anti-CD25, CXCR3, CCR4, and CD103 antibodies. Microbeads were added before surface staining to calculate cell numbers. All antibodies were purchased from eBiosciences except as indicated.

Colonic biopsies were kept in 0.9% NaCl solution at 4°C overnight before being preprocessed into fine pieces, followed by gentle rotation in a 37°C water bath for 45 min in Hank’s balanced salt solution (Ca^2+^, Mg^2+^ free) supplemented with 10 mM HEPES (pH 7.2), 5% fetal bovine serum, 100 U/ml penicillin, 100 µg/ml streptomycin, and 5 mM EDTA. Intraepithelial lymphocytes (IELs) were recovered by centrifugation, activated with PMA (100 ng/ml) and ionomycin (1 µM) in the presence of monensin (2 µM) for 5 h. Cytokine expression of CD8^+^ T cells were analyzed by surface and intracellular staining with anti-CD45, CD3, CD4, CD8a, γδ-TCR, and IFN-γ and IL-4 antibodies, respectively.

### Immunofluorescence Staining of Colonic Biopsies

Paraffin-embedded sections of colonic biopsies were deparaffinized in xylene, rehydrated in decreasing concentrations of ethanol, and then heated in 0.01 M citric acid buffer (pH 6.0) for 15 min at 95°C to reveal antigens. After deparaffinization, sections were rinsed in blocking buffer (1× PBS/5% normal goat serum/0.3% Triton X-100) for 1 h at room temperature (RT) and then incubated sequentially with rabbit anti-human CD8 (1:100) (Abcam) overnight at 4°C, goat anti-rabbit-IgG-Cy3 (1:500) (Abcam) for 1 h at RT, rabbit anti-human cleaved (Asp175) caspase-3 (Cell Signaling Technology) overnight at 4°C, goat anti-rabbit-IgG-Alexa Fluor 488 (1:500) (Abcam) for 1 h at RT, and anti-human IL-4-APC (BioLegend) overnight at 4°C. The sections were mounted with a Vector Shield mounting solution containing 4′,6-diamidino-2-phenylindole (H-1200, Vector). Washing three times with 1× PBS was performed between incubations. Immunofluorescence images were acquired with a Leica TCS SP8 confocal system (Leica Microsystems) using a 20×/0.75 dry objective lens and analyzed with Leica X image analysis software (Leica). Post-acquisition processing (brightness, opacity, contrast, and color balance) was applied to the entire image and accurately reflects that of the original.

### Metabolic Assays

To measure glucose uptake, cells were first cultured in glucose-free media for 2 h, followed by 30 min incubation at 37°C with 30 µM 2-NBGD (Life Technologies) and flow cytometry analysis. The Glycolytic Cell-Based Assay Kit (Cayman Chemicals) was used to measure lactate secreted into the medium. Cells from day 4 cultures were incubated at 5 × 10^5^/ml in fresh IP5 media for 20 h. To measure mitochondria membrane potential, cells were incubated at 37°C for 20 min with 100 nM Mitotracker Orange (Life Technologies), which stains mitochondria in live cells, and its accumulation, measured by flow cytometry, is dependent on membrane potential.

### Metabolic Extraction and Gas Chromatography–Mass Spectrometry

CD8^+^ T cells (~0.5 × 10^6^) that had been activated for 4 days in the presence of IL-2 or IL-6 and TGF-β as above were rapidly cooled in ice-cold PBS and centrifuged at 4°C. Chloroform (50 µl) was added to the cell pellet, followed by extraction with methanol: water (3:1, 200 µl) containing stable isotopically labeled internal standards (^13^C_6_-sorbitol, ^13^C_5_, ^15^N-valine). The samples were placed on ice for 10 min with intermittent vortexing, then centrifuged to pellet cell debris and proteins (14,000 rpm, 5 min, 4°C). The supernatant was transferred to a clean tube containing Milli-Q water (100 µl), vortexed, then centrifuged (14,000 rpm, 5 min, 4°C) to promote biphasic partitioning. The upper aqueous phase containing polar metabolites was evaporated to dryness under vacuum and chemically derivatized for analysis by gas chromatography–mass spectrometry at Metabolomics Australia (Melbourne, VIC, Australia). Derivatization, GC/MS analysis, and data processing were performed as previously described (15). After GC/MS peaks were annotated and quantified, intensities (area under curve) were log-transformed and median-normalized. For metabolites with multiple detectable peaks, all measurements were summed up for representation. The Student’s *t*-test was performed to compare metabolite abundances of the different treatments and *P*-values adjusted for multiple testing using the Benjamini–Hochberg method (16).

### RNA-Seq Analysis

Total RNA was isolated from naïve and day 4-activated CD8^+^ T cells with the RNeasy Mini Kit (QIAGEN). Libraries were constructed as per the Illumina TruSeq Stranded Total RNA Sample Preparation kit and sequenced with Illumina NextSeq 500. RNA-Seq data were quality assessed by FastQC (0.10.1).^1^ Residual adapter sequences were removed from reads and bases with a Phred score <25 trimmed from the start and end of reads. Reads < 50 bp long were discarded (Trimmomatic [0.33]) (17). Trimmed reads were mapped to the human genome (hg19) using STAR (2.4.0h1) in two-pass mode with default parameters (18). Duplicates were removed using MarkDuplicates (1.99 [1563]) from Picard tools.^2^ Read counts were then summarized across genes (GENCODE v19) using featureCounts (1.4.6) (19).

Raw read counts were imported into the R (3.3.1) statistical computing environment and analyzed using functions provided by the *limma* (3.30.13) (20) and *edgeR* (3.16.5) Bioconductor (3.4) packages. Genes with less than one count per million (cpm) in at least ten samples were removed from subsequent analysis. Counts were then normalized using trimmed mean of *M*-values (21). The *limma* “voom” (22) function was applied to the normalized counts to estimate the mean–variance relationship and generate precision weights for each observation, ready for linear modeling.

Gene-wise linear models were fitted to the “voom”-transformed log_2_ cpm to determine differences in gene expression between activated and naive CD8^+^ T cells, accounting for individual to individual variation. Statistically significant differentially expressed genes were identified using empirical Bayes moderated *t*-tests (23), allowing for a mean–variance trend and performing robust empirical Bayes shrinkage of the gene-wise variances to protect against hypervariable genes (24). *P*-values were adjusted for false discovery rate using the Benjamini–Hochberg method (16). KEGG pathways from the C2 curated gene sets in the Broad Institute Molecular Signatures Database were interrogated using the “camera” competitive gene set testing method (25), which tests whether a set of genes is highly ranked relative to other genes in terms of differential expression, accounting for inter-gene correlation.

### Statistics

Statistics was performed with Prism 6 software (GraphPad Software). For paired comparisons, a *P*-value was calculated by Wilcoxon matched-pairs signed rank test. For unpaired comparisons, *P*-values were determined by Mann–Whitney *U* test. Correlations were determined by linear regression. *P*-values for multiple comparisons were adjusted by the Benjamini–Hochberg method (16).

## Results

### Naïve Cord Blood CD8^+^ T Cells Respond Distinctly to Lineage Differentiation Cytokines

**Figure 1:**
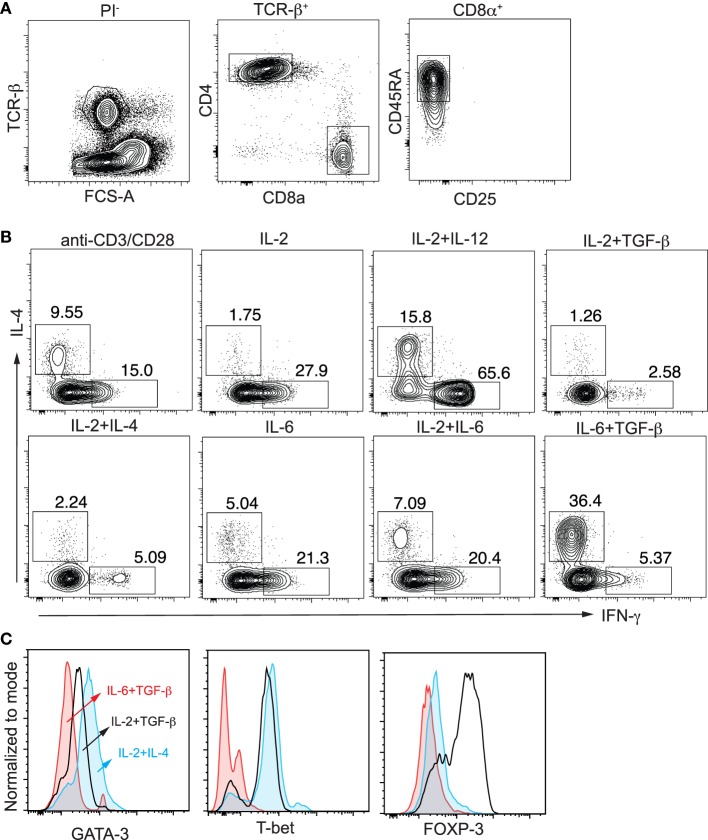
Differentiation of naïve cord blood CD8^+^ T cells. **(A)** Flow sorting of naïve CD8^+^ T cells from thawed cord blood mononuclear cells; **(B)** intracellular expression of IFN-γ and IL-4 after activation of naïve CD8^+^ T cells with anti-CD3/CD28 microbeads (1:1) ± cytokines; **(C)** decreased expression of GATA-3 and T-bet after induction of IL-4 expression by IL-6 + TGF-β in CD8^+^ T cells compared with cells activated by IL-2 + IL-4 and IL-2 + TGF-β. Data are representative of *n* > 10 donors, all showing similar results.

We first examined cytokine and transcription factor expression in flow-sorted naïve cord blood CD8^+^ T cells (Figure 1A) when they were activated under CD4^+^ T_H_ differentiation conditions. In the presence of IL-2 ± IL-12, IL-6 + TGF-β, or IL-2 + TGF-β CD8^+^ T cells expressed IFN-γ, IL-4, or FOXP3, respectively (Figures 1B,C). IL-2 + IL-4 did not induce significant IL-4 expression, as for classic Th2 cells. Of note, IL-6 + TGF-β-induced IL-4 expression was associated with decreased expression GATA-3 and T-bet (Figure 1C). Thus, cord blood CD8^+^ T cells differentiate under IL-12/T_H_1 and IL-2 + TGFβ/iTreg conditions similarly to CD4^+^ T cells, as we have shown previously (13). However, in contrast to classic Th2 differentiation, cord blood CD8^+^ T cells differentiated into IL-4 -expressing “T_C_2” cells in the presence of IL-6 + TGF-β, not IL-2 + IL-4, and this process was associated with decreased expression of the classic Th2 transcription factor GATA-3.

### IL-2 Suppresses and TGF-β Promotes Cord Blood T_C_2 Differentiation

**Figure 2:**
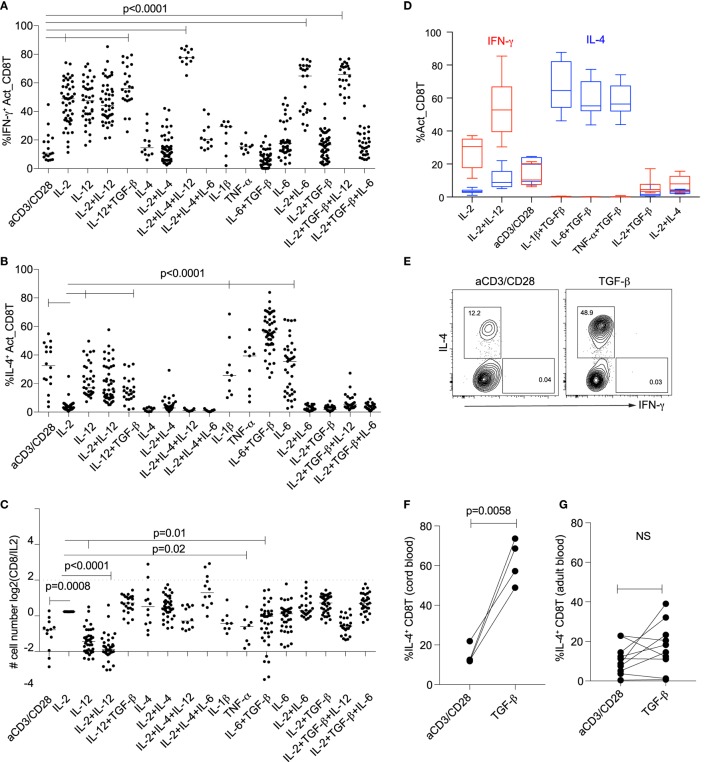
Differentiation of naïve cord blood CD8^+^ T cells is modified by different combinations of cytokines. Differentiation of cord blood CD8^+^ T cells activated with anti-CD3/CD28 microbeads (1:1) ± cytokines in different individuals. Proportions of CD8^+^ T cells expressing **(A)** IFN-γ or **(B)** IL-4 after cells were activated in the presence of different cytokines. **(C)** Numbers of cells generated when naïve CD8^+^ T cells were activated in the presence of different cytokines, relative to IL-2 alone. *P*-values between indicated groups were calculated by Kruskal–Wallis test and corrected for multiple comparison with Original FDR method of Benjamini and Hochberg. **(D)** Proportions of IFN-γ and IL-4-expressing CD8^+^ T cells after activation in the presence of inflammatory cytokines (*n* = 10). Effects of activation and TGF-β on IL-4 expression for cord blood **(E,F)** and adult **(G)** CD8^+^ T cells. *P*-values calculated by paired *t*-test.

After activation *via* the CD3 and CD28, a small proportion (median 11%) of cord blood naïve CD8^+^ T cells expressed IFN-γ (Figure 2A). The proportion of CD8^+^ T cells expressing IFN-γ increased when cells were activated in the presence of IL-2 or IL-12, separately or together, or in combination with a range of other cytokines (Figure 2A), consistent with the key roles of IL-2 and IL-12 in Tc1 differentiation. The dominant effect of IL-12 on IFN-γ expression was further evident in that it overcame suppression of IL-2-induced IFN-γ expression under classical CD4^+^ T_H_2 (IL-2 + IL-4) or iTreg (IL-2 + TGF-β) conditions (Figure 2A).

High proportions of IL-4 expression were observed when CD8^+^ T cells were activated in the absence of cytokines, or in the presence of IL-1β ± TGF-β, TNF-α ± TGF-β or IL-6 ± TGF-β, compared with in the presence of IL-2 (Figures 2B,D). Substantial IL-4 expression was also observed when CD8^+^ T cells were activated by IL-12 ± IL-2 and IL-12 ± TGF-β (Figure 2B). Generally, the number of cells recovered at day 5, when CD8^+^ T cells were activated in the absence of cytokines, or in the presence of IL-12, IL-6 + TGF-β, was significantly decreased, compared with cells recovered in the presence of IL-2 (Figure 2C).

Given that cord blood CD8^+^ T cells expressed IFN-γ, FOXP3, or IL-4 when they were activated by combinations of IL-12, IL-2, or IL-1β/IL-6/TNF-α with TGF-β, respectively, we analyzed the contributions of TGF-β alone to IL-4 expression in CD8^+^ T cells. Expression of IL-4 was significantly increased when cord blood CD8^+^ T cells were activated in the presence of TGF-β (Figures 2E,F). By contrast, TGF-β alone did not increase IL-4 expression in naïve CD8^+^ T cells (isolated from buffy coat) from adult donors (Figure 2G).

### TGF-β Induces Expression of Mucosa-Chemotactic Molecules on Differentiated CD8^+^ T Cells

**Figure 3:**
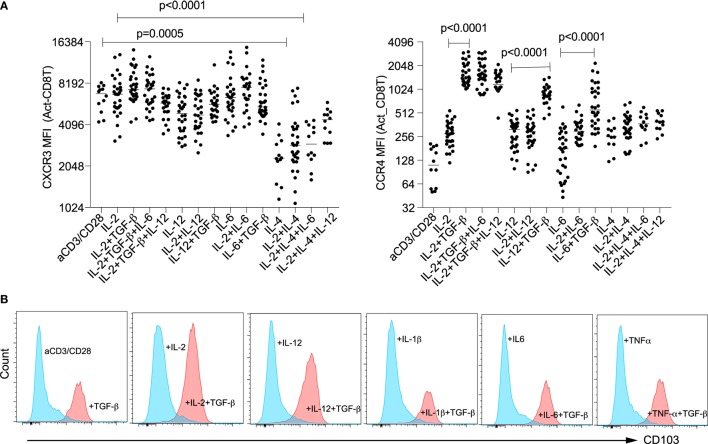
TGF-β induces expression of mucosa-chemotactic molecules on CD8^+^ T cells. Surface expression of chemokine receptors CXCR3 and CCR4 **(A)** and integrin CD103 **(B)** 4 days after activation of naïve CD8^+^ T cells under specified conditions (data are representative of *n* > 10 donors). *P*-values between indicated groups were calculated on matched pairs by Wilcoxon matched-pairs signed rank.

TGF-β has traditionally been regarded as an anti-inflammatory cytokine, as evidenced by its role to promote regulatory CD4^+^ and CD8^+^ T cell development in the presence of IL-2. However, we previously found that TGF-β, in combination with IL-1-β or TNF-α, induced non-classic CD4^+^ “T_H_2”/IL-4 cells from naïve cord blood CD4^+^ T cells (13). In cord blood CD8^+^ T cells, TGF-β induced T_C_1 development in combination with IL-12, but TGF-β alone, or in combinations with IL-1β/TNF-α/IL-6 induced “T_C_2” development (Figure 2). TGF-β is expressed by many cell types, including constitutively by lung stromal and gut epithelia cells involved in allergic asthma and food allergy, and is chemo-attractive for innate lymphoid type 2 cell trafficking (26). We therefore further investigated a role for TGF-β in CD8^+^ T cell trafficking. First, we examined whether the chemokine receptors CXCR3 and CCR4 expressed by CD4^+^ T_H_1 or T_H_2 cells, respectively, were present on CD8^+^ T cells that express IFN-γ or IL-4. Expression of CXCR3 was decreased by IL-4 (Figure 3A; left panel). On the other hand, TGF-β consistently induced expression of CCR4 (Figure 3A; right panel) and alpha E integrin (CD103) (Figure 3B), irrespective of the CD8^+^ T cell phenotype.

### CD8^+^ T Cell Differentiation Is Conditioned by IL-2-IL-2R Signaling

**Figure 4:**
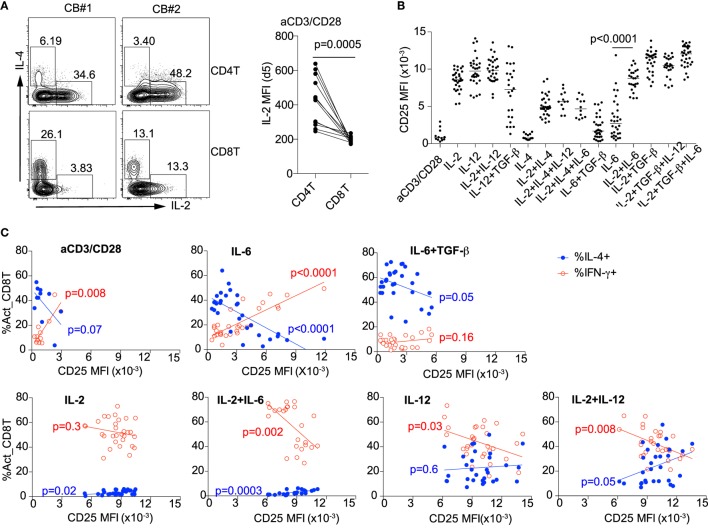
CD8^+^ T cell differentiation is determined by IL-2–IL-2R signaling. **(A)** Expression of IL-2 in activated CD4^+^ and CD8^+^ T cells at day 5. **(B)** Expression of IL-2 receptor alpha chain CD25 on day 4 after activation of CD8^+^ T cells in the presence of different cytokines. **(C)** Under anti-CD3/CD28, IL-6 and IL-6 ± TGF-β T_C_2 conditions, decreased expression of CD25 correlates with increased expression of IL-4 (upper panel); under IL-2 + IL-6, IL-12, IL-2 + IL-12 T_C_1 conditions, decreased expression of CD25 correlates with increased expression of IFN-γ (lower panel).

Previously, we found that suppression of IL-2 by inflammatory cytokines and TGF-β was required for IL-4 expression by activated cord blood CD4^+^ T cells (13). In contrast to CD4^+^ T cells, activated cord blood CD8^+^ T cells expressed significantly less IL-2 (Figure 4A). This suggests that cord blood CD8^+^ T cells may be poised to default toward “T_C_2” differentiation. Because IL-2 was critical for induction of T_C_1 but suppressed “T_C_2” differentiation, we examined the relationship between IL-2 receptor expression and CD8^+^ T cell differentiation. Expression of the IL-2 receptor alpha chain (IL-2RA) (CD25) was increased in the presence of IL-2 and/or IL-12 but not IL-6 ± TGF-β “T_C_2” (IL-4) conditions (Figure 4B). When naïve CD8^+^ T cells from different individuals were activated alone or under “T_C_2” (IL-6) conditions, the decrease in CD25 expression was associated with an increase in IL-4 and decrease in IFN-γ expression. The reciprocal association of CD25 and IL-4 persisted under IL-6 + TGF-β conditions (Figure 4C). By contrast, under T_C_1 (IL-2 + IL-6, IL-12, or IL-2 + IL-12) conditions, the decrease in CD25 expression was associated with the increase in IFN-γ expression (Figure 4C). These findings illustrate how intrinsic IL-2 receptor signaling differentially regulates T cell differentiation in cord blood according to the cytokine milieu.

### Differentiation of “T_C_2” Cells Is Associated With Decreased Glycolysis

**Figure 5:**
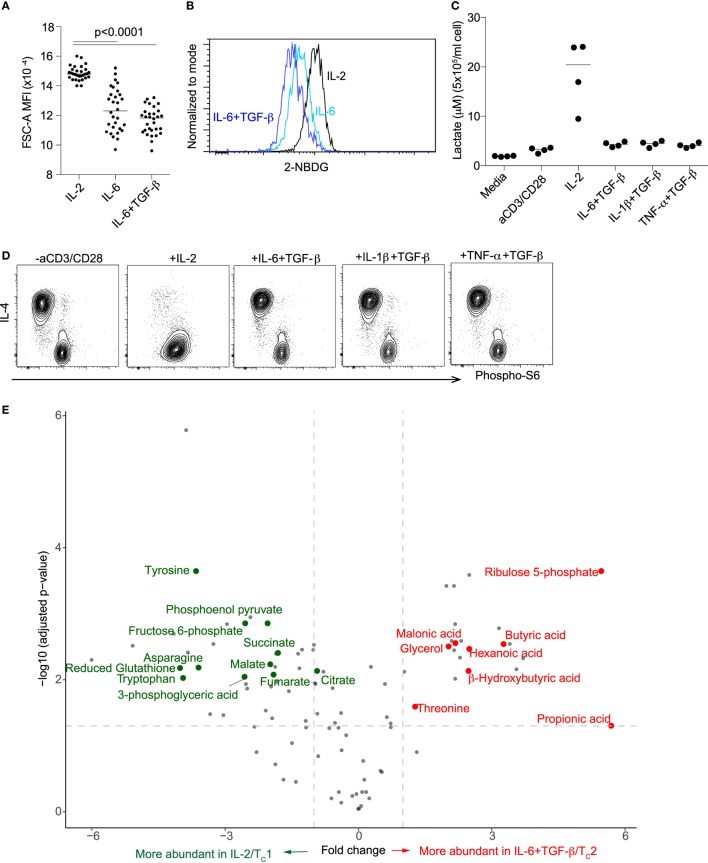
Differentiation of “T_C_2” cells is associated with decreased glycolysis. Cell size **(A)**, glucose uptake **(B)** and secretion of lactate **(C)** after activation of CD8^+^ T cells under T_C_1 or “T_C_2” conditions. All parameters of glycolysis were decreased under “T_C_2” conditions. **(D)** Decreased phosphorylation of S6, an mTOR substrate, associated with expression of IL-4. **(E)** Metabolites in CD8^+^ T cells that were activated under T_C_1/IL-2 and IL-6 + TGF-β/“T_C_2” conditions. Data are representative of three independent experiments.

T_C_1 activation is known to be associated with an increase in aerobic glycolysis, dependent on IL-2 (27–29). On the other hand, naïve and memory T cell activation have been linked to fatty acid oxidation (30). To investigate the metabolic program under “T_C_2” conditions, we measured cell size, glucose uptake, lactate secretion and phosphorylation of the mTOR substrate ribosomal protein S6, each known to increase with glycolytic activity (31). In CD8^+^ T cells activated under “T_C_2” compared with IL-2/T_C_1 conditions all these parameters of glycolysis were decreased (Figures 5A–D). Notably, IL-4-positive cells had the lowest expression of phosphorylated S6 compared with IL-4-negative cells (Figure 5D). Inhibition of glycolysis was also confirmed by direct measurement of intracellular metabolites. At day 4, compared with activation in the presence of IL-2, CD8^+^ T cells activated in the presence of IL-6 + TGF-β had decreased intermediary glycolytic (fructose-6-phosphate, 3-phosophoglycerate, phosphoenol pyruvate) and tricarboxylic acid cycle (citrate, fumarate, and malate) metabolites. Downregulation of citrate, a biosynthetic precursor of fatty acid synthesis (FAS), suggests that IL-4 expression in CD8^+^ T cells might depend on an alternative energy source. Indeed, the concentrations of short-chain fatty acids (SCFAs), propionic acid, butyric acid, β-hydroxybutyric acid, and hexanoic acid were significantly increased with IL-6 + TGF-β, indicating a shift in fatty acid oxidation (Figure 5E).

### “T_C_2” Differentiation Is Associated With Increased Expression of Lipid Metabolism Genes and Is Caspase Dependent

**Figure 6:**
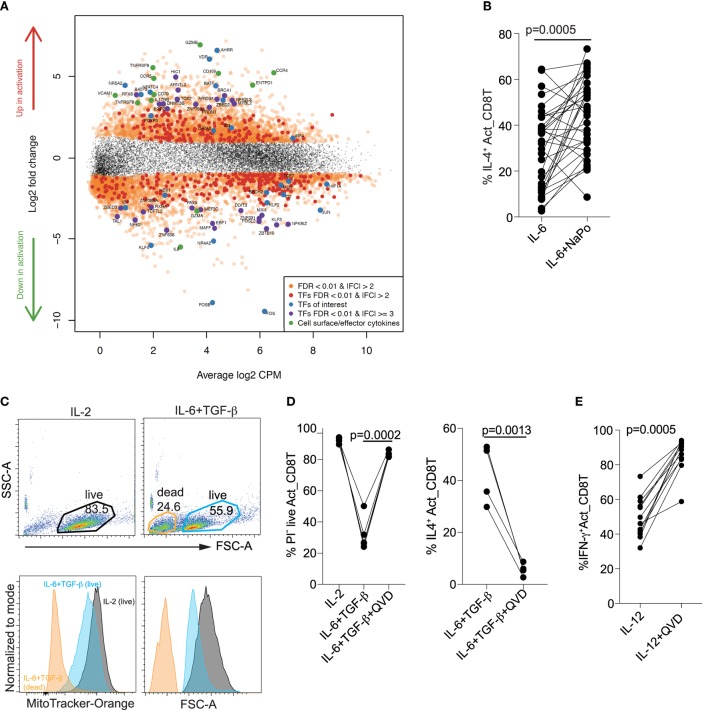
Differentiation of “T_C_2” cells is associated with increased fatty acid metabolism and is caspase dependent. **(A)** Mean-difference plot showing changes in gene expression associated with “T_C_2” differentiation. **(B)** IL-4 expression is increased with supplementation of sodium propionate (NaPo) (right). **(C)** Decreased mitochondrial membrane potential (MitoTracker-Orange stain) and cellular size in live “T_C_2” compared with T_C_1 cells. Data are representative of *n* > 10 donors, all with similar results. **(D)** Prevention of cell death and IL-4 expression at day 5 by the caspase inhibitor QVD-Oph added at day 0 and supplemented at day 3. **(E)** Enhancement of IFN-γ expression at day 5 by the caspase inhibitor QVD-Oph added at day 0 and supplemented at day 3. *P*-values were calculated by Wilcoxon matched-pairs signed rank test.

To gain insight into mechanisms underlying “T_C_2” differentiation, we examined transcriptional profiles by RNA-Seq of naïve cord blood CD8^+^ T cells before and 4 days after activation in the presence of IL-6 + TGF-β (Figure S1 and Table S1 in Supplementary Material). Four hundred fifty-seven genes were significantly (FDR < 1%) differentially expressed by more than twofold in either direction. The decreased expression of *MYC* (27) and *HIF1A* (32) is consistent with inhibition of glycolysis. For several reasons, the increased expression of *AHRR, BATF3*–*BATF*–*IRF4*, and the decreased expression of *BACH2*, could also be consistent with the “T_C_2” phenotype. Thus, the product of *AHRR* (aryl hydrocarbon receptor repressor) is highly expressed in immune cells of the skin and intestine and its expression in mouse CD4^+^ T cells is induced by IL-6 + TGF-β (33). The BATF–IRF4 complex binds to AP-1 motifs and augments IL-4 expression, while BACH2–BATF antagonizes the recruitment of BATF–IRF4. In the mouse, IL-4 increases the expression of *Batf* and *Irf4* and decreases the expression of *Bach2* (34) (Figure 6A). Although *GATA3* mRNA was increased at day 4, we did not observe any increase in its protein expression by day 5 when IL-4 was expressed.

To identify signaling pathways associated with “T_C_2” differentiation, we examined whether KEGG pathways from the Broad Institute Curated gene sets were enriched for differential expression (Table S2 in Supplementary Material). We observed significant enrichment of upregulated genes in pathways involved in fatty acid metabolism (Figure S2 in Supplementary Material). This was consistent with the increase in intracellular SCFAs as described earlier (see Figure 5E). The ribosome biogenesis pathway was, however, significantly enriched for downregulated genes (Figure S3 in Supplementary Material). Given that colonic bacteria ferment undigested complex carbohydrates to SCFAs, we postulated that with limited IL-2 signaling and decreased glycolysis, CD8^+^ T cells would utilize SCFAs to produce IL-4. Indeed, supplementation with the SCFAs sodium propionate (NaPo) increased IL-4 expression when naïve CD8^+^ T cells were activated in the presence of IL-6 (Figure 6B).

Disruption of ribosome biogenesis and protein translation is known to inhibit CD8^+^ T cell proliferation (35) and promote caspase-dependent cell death (36). We consistently observed decreased cell numbers under “T_C_2” conditions (see Figure 2C). This was in part due to increased cell death when CD8^+^ T cells were activated under “T_C_2” conditions. In fact, at day 5 CD8^+^ T cells activated and differentiated to “T_C_2” cells in the presence of IL-6 + TGF-β were smaller and had reduced mitochondrial membrane potential compared with CD8 ^+^ T cells activated with IL-2 (Figures 6C,D). The pan-caspase inhibitor QVD-Oph prevented cell death and, surprisingly, also completely inhibited IL-4 expression (Figure 6D). The caspase-dependent expression of IL-4 was specific as IFN-γ expression was not decreased but rather increased with IL-12 in the presence of QVD-Oph (Figure 6E).

### “T_C_2” Differentiation Is Increased in Colonic Mucosa of Children With Idiopathic Colitis

**Figure 7:**
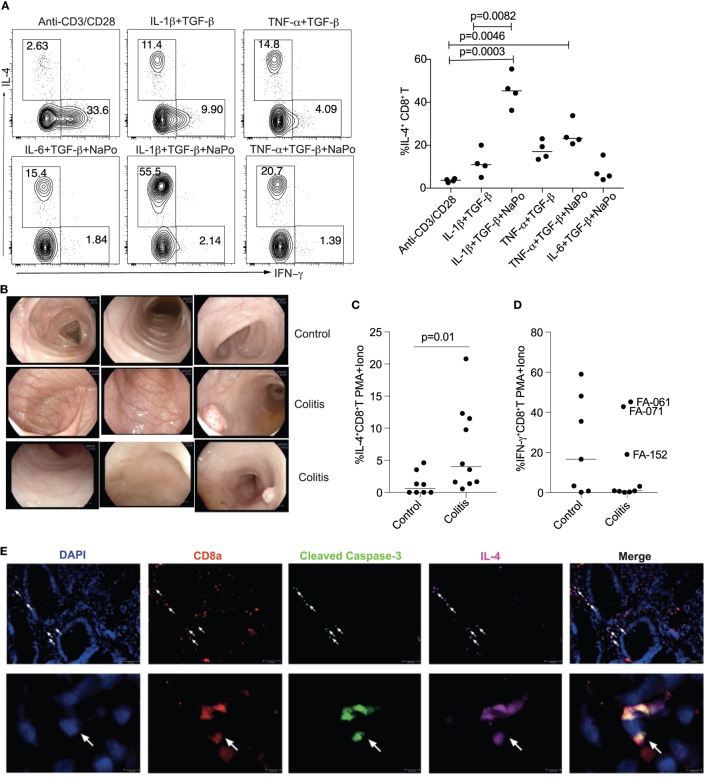
“T_C_2” differentiation is increased in colonic mucosa of children with colitis. **(A)** “T_C_2” differentiation in naïve CD8^+^ T cells isolated from children with non-inflammatory bowel disease and non-infectious pediatric idiopathic colitis (PIC). **(B)** Representative endoscopic images of colonic mucosa from control children and children with PIC. **(C,D)** Intracellular expression of IL-4 and IFN-γ in CD8^+^ intraepithelial lymphocytes from colonic mucosa following stimulation with PMA and ionomycin. *P*-value was calculated by Mann–Whitney *U* test. **(E)** Expression *in situ* of CD8, activated caspase-3, and IL-4 in paraffin-embedded sections of colonic biopsies. Data are representative of *n* > 3 independent experiments.

To obtain evidence that inflammation could promote differentiation of non-classic “T_C_2” cells *in vivo*, we first examined if naïve CD8^+^ T cells from blood of children with idiopathic colitis were able to produce IL-4 under conditions that mimic colonic inflammation. We found that TGF-β and NaPo, together with IL-1β or TNF-α, promoted IL-4 expression in CD8^+^ T cells *in vitro* (Figure 7A). We then measured IL-4 and IFN-γ secretion by colonic mucosal IELs derived from colonic biopsies, 10 of which were reported as non-IBD and non-infectious PIC and 8 as normal (Table S3 in Supplementary Material; Figure 7B). After activation of IELs with PMA and ionomycin, expression of IL-4 was significantly increased in PIC compared with controls (Figure 7C), but IFN-γ expression was not statistically different between the groups (Figure 7D). To relate IL-4 expression with caspase-dependent cell death, we performed immunofluorescence staining on formalin-fixed, paraffin-embedded sections of colonic biopsies, which revealed co-expression of IL-4 with activated caspase-3 in CD8^+^ T cells (Figure 7E).

In summary, induction of IL-4 expression in cord blood CD8^+^ T cells is associated with suppression of IL-2 receptor signaling and glycolysis, increased fatty acid metabolism, caspase activation and cell death. These findings provide a plausible explanation for the appearance of CD8^+^IL-4^+^ IELs present in children with colitis.

## Discussion

Consistent with classical patterns of cytokine-induced Th1 and Treg differentiation as observed in CD4^+^ T cells, naïve cord blood CD8^+^ T cells differentiated into T_C_1 and T_CREG_ lineages characterized by expression of IFN-γ and FOXP3, respectively. By contrast, IL-4 failed to induce classical IL-4-expressing (T_C_2) CD8^+^ T cells. However, substantial IL-4 expression was observed when cord blood CD8^+^ T cells were activated alone, with TGF-β, or combinations of inflammatory cytokines (IL-1β/IL-6/TNF-α, IL-12) with TGF-β or NaPo. These non-classical, IL-4-expressing CD8^+^ T cells displayed decreased glycolysis but increased expression of the genes associated with fatty acid metabolism. Intriguingly, expression of IL-4 was caspase dependent and associated with increased cell death. These findings mirror those we reported for cord blood CD4^+^ T cells, after observing that infants who developed food allergy had an increase in inflammatory cytokine expression by activated monocytes in their cord blood (13).

IL-2 is produced mainly by activated CD4^+^ T cells and, *via* the high affinity IL-2R, maintains Myc (37) expression in CD4^+^ and CD8^+^ T cells, enhances transcription of genes for glycolysis, and promotes T_H_1 and T_C_1 differentiation (38). Limiting IL-2 signaling upregulates expression of Bcl6, inhibits glycolysis (29), and results in loss of mitochondria membrane potential and caspase-dependent cell death (39). The release of self-antigens (e.g., DNA) from apoptotic cells may elicit unwanted inflammation. In this regard, activation of caspases 9, 3, and 7 following apoptotic cell death has been shown to actively suppress inflammation (40). It is conceivable that activated caspases in pre-apoptotic CD8^+^ T cells might also unleash a yet to be defined translational suppression mechanism of IL-4 (41). In fact, caspase-dependent expression of IL-4 could represent a default mechanism for apoptosis-associated immune suppression through the differentiation of anti-inflammatory macrophages and inhibition of type 1 immune responses (42). Indeed, IL-4 expression has been previously observed in T cells, monocytes, neutrophils and bronchial epithelial cells undergoing cell death (43–45). In the face of elevated or sustained inflammatory conditions, over-expression of IL-4 may, however, result in immune pathology. In fact, we found that IL-4 expression by colonic CD8^+^ IELs was significantly increased in children with colitis compared with control subjects. Furthermore, cell death may promote the release of DNA and associated self-antigens, leading to the IL-4 dependent generation of IgE-specific autoantibodies, as described in systemic lupus erythematosus (46). Interestingly, administration of low-dose IL-2 markedly decreased disease severity in patients with SLE (47). Our demonstration that limiting IL-2 signaling is necessary for caspase-dependent IL-4 expression, suggests that administration of IL-2 might potentially alleviate immune-allergic diseases in children.

## Ethics Statement

This study was carried out in accordance with the recommendations of the International Ethical Guidelines for Research Involving Human Subjects. The protocols were approved by the Human Ethics Committees of Walter and Eliza Hall Institute, Barwon Health, Geelong, and Guangzhou Women and Children’s Medical Center (GWCMC). Legal guardians of all subjects gave written informed consent in accordance with the Declaration of Helsinki. Cord blood and colon biopsies were obtained from Barwon Infant Study (BIS) (14) and hospitalized children at GWCMC (Ethics Number 2017072601). Children (*n* = 18) at GWCMC underwent colonoscopy and biopsy for the diagnostic evaluation of chronic abdominal pain, vomiting, or lower gastrointestinal bleeding (Table S3 in Supplementary Material). Colitis was identified endoscopically as having mucosal swelling, hyperemia, erosion, or ulceration and histologically as impaired epithelial integrity, increased lymphocytes and/or eosinophil [>20/HPF (high power field)] infiltrations. Control colonic biopsies were from children who displayed no features of colitis on colonoscopy and histology.

## Author Contributions

YZ and LH conceived the ideas, analyzed the data, and wrote the manuscript. PV and NH provided cord blood samples. YZ, GN, BH, LZ, and YX performed the experiments. HC, SG, and MY provided clinical care, performed colonoscopies, and characterized children with colitis. JM and AO performed bioinformatic analysis for RNA-Seq. KW provided assistance with statistics. DS and HL analyzed metabolic data. JMW and AK provided assistance in metabolic assays and interpretation of the data. All the authors discussed and approved the manuscript.

## Conflict of Interest Statement

The authors declare that the research was conducted in the absence of any commercial or financial relationships that could be construed as a potential conflict of interest.

## Footnotes

^1^http://www.bioinformatics.babraham.ac.uk/projects/fastqc.

^2^https://github.com/broadinstitute/picard.

## References

[1] Zhu J Yamane H Paul WE. Differentiation of effector CD4 T cell populations (*). Annu Rev Immunol (2010) 28:445–89. 10.1146/annurev-immunol-030409-101212 20192806 PMC3502616

[2] Newton K Dixit VM. Signaling in innate immunity and inflammation. Cold Spring Harb Perspect Biol (2012) 4. 10.1101/cshperspect.a006049 22296764 PMC3282411

[3] Croft M Carter L Swain SL Dutton RW. Generation of polarized antigen-specific CD8 effector populations: reciprocal action of interleukin (IL)-4 and IL-12 in promoting type 2 versus type 1 cytokine profiles. J Exp Med (1994) 180:1715–28. 10.1084/jem.180.5.1715 7525836 PMC2191720

[4] Srenathan U Steel K Taams LS. IL-17+ CD8+ T cells: differentiation, phenotype and role in inflammatory disease. Immunol Lett (2016) 178:20–6. 10.1016/j.imlet.2016.05.001 27173097 PMC5046976

[5] Leong YA Chen Y Ong HS Wu D Man K Deleage C CXCR5(+) follicular cytotoxic T cells control viral infection in B cell follicles. Nat Immunol (2016) 17:1187–96. 10.1038/ni.3543 27487330

[6] Mahic M Henjum K Yaqub S Bjornbeth BA Torgersen KM Tasken K Generation of highly suppressive adaptive CD8(+)CD25(+)FOXP3(+) regulatory T cells by continuous antigen stimulation. Eur J Immunol (2008) 38:640–6. 10.1002/eji.200737529 18266270

[7] Kollmann TR Levy O Montgomery RR Goriely S. Innate immune function by toll-like receptors: distinct responses in newborns and the elderly. Immunity (2012) 37:771–83. 10.1016/j.immuni.2012.10.014 23159225 PMC3538030

[8] Reynaldi A Smith NL Schlub TE Venturi V Rudd BD Davenport MP. Modeling the dynamics of neonatal CD8+ T-cell responses. Immunol Cell Biol (2016) 94(9):838–48. 10.1038/icb.2016.47 27142943 PMC5069106

[9] Liao SY Liao TN Chiang BL Huang MS Chen CC Chou CC Decreased production of IFN gamma and increased production of IL-6 by cord blood mononuclear cells of newborns with a high risk of allergy. Clin Exp Allergy (1996) 26:397–405. 10.1046/j.1365-2222.1996.d01-325.x 8732236

[10] Prescott SL Noakes P Chow BW Breckler L Thornton CA Hollams EM Presymptomatic differences in toll-like receptor function in infants who have allergy. J Allergy Clin Immunol (2008) 122:391–9, 399.e1–5. 10.1016/j.jaci.2008.04.042 18571707

[11] Tulic MK Hodder M Forsberg A Mccarthy S Richman T D’Vaz N Differences in innate immune function between allergic and nonallergic children: new insights into immune ontogeny. J Allergy Clin Immunol (2011) 127:470–8.e1. 10.1016/j.jaci.2010.09.020 21093030

[12] Prescott SL Macaubas C Smallacombe T Holt BJ Sly PD Holt PG. Development of allergen-specific T-cell memory in atopic and normal children. Lancet (1999) 353:196–200. 10.1016/S0140-6736(98)05104-6 9923875

[13] Zhang Y Collier F Naselli G Saffery R Tang ML Allen KJ Cord blood monocyte-derived inflammatory cytokines suppress IL-2 and induce nonclassic “TH2-type” immunity associated with development of food allergy. Sci Transl Med (2016) 8:321ra8 10.1126/scitranslmed.aad4322 26764159

[14] Vuillermin P Saffery R Allen KJ Carlin JB Tang ML Ranganathan S Cohort profile: the Barwon Infant Study. Int J Epidemiol (2015) 44:1148–60. 10.1093/ije/dyv026 25829362

[15] Overgaard AJ Weir JM De Souza DP Tull D Haase C Meikle PJ Lipidomic and metabolomic characterization of a genetically modified mouse model of the early stages of human type 1 diabetes pathogenesis. Metabolomics (2016) 12:13. 10.1007/s11306-015-0889-1 26612984 PMC4648980

[16] Benjamini Y Hochberg Y Controlling the false discovery rate: a practical and powerful approach to multiple testing. J R Stat Soc Series B Stat Methodol (1995) 57:289–300.

[17] Bolger AM Lohse M Usadel B. Trimmomatic: a flexible trimmer for Illumina sequence data. Bioinformatics (2014) 30:2114–20. 10.1093/bioinformatics/btu170 24695404 PMC4103590

[18] Dobin A Davis CA Schlesinger F Drenkow J Zaleski C Jha S STAR: ultrafast universal RNA-seq aligner. Bioinformatics (2013) 29:15–21. 10.1093/bioinformatics/bts635 23104886 PMC3530905

[19] Liao Y Smyth GK Shi W. featureCounts: an efficient general purpose program for assigning sequence reads to genomic features. Bioinformatics (2014) 30:923–30. 10.1093/bioinformatics/btt656 24227677

[20] Ritchie ME Phipson B Wu D Hu Y Law CW Shi W limma powers differential expression analyses for RNA-sequencing and microarray studies. Nucleic Acids Res (2015) 43:e47. 10.1093/nar/gkv007 25605792 PMC4402510

[21] Robinson MD Oshlack A. A scaling normalization method for differential expression analysis of RNA-seq data. Genome Biol (2010) 11:R25. 10.1186/gb-2010-11-3-r25 20196867 PMC2864565

[22] Law CW Chen Y Shi W Smyth GK. Voom: precision weights unlock linear model analysis tools for RNA-seq read counts. Genome Biol (2014) 15:R29. 10.1186/gb-2014-15-2-r29 24485249 PMC4053721

[23] Smyth GK Limma: linear models for microarray data. In: Gentleman R Carey VJ Huber W Irizarry RA Dudoit S, editors. Bioinformatics and Computational Biology Solutions Using R and Bioconductor. Statistics for Biology and Health. New York, NY: Springer (2005). p. 397–420.

[24] Phipson B Lee S Majewski IJ Alexander WS Smyth GK. Robust hyperparameter estimation protects against hypervariable genes and improves power to detect differential expression. Ann Appl Stat (2016) 10:946–63. 10.1214/16-AOAS920 28367255 PMC5373812

[25] Wu D Smyth GK. Camera: a competitive gene set test accounting for inter-gene correlation. Nucleic Acids Res (2012) 40:e133. 10.1093/nar/gks461 22638577 PMC3458527

[26] Denney L Byrne AJ Shea TJ Buckley JS Pease JE Herledan GM Pulmonary epithelial cell-derived cytokine TGF-beta1 is a critical cofactor for enhanced innate lymphoid cell function. Immunity (2015) 43:945–58. 10.1016/j.immuni.2015.10.012 26588780 PMC4658339

[27] Wang R Dillon CP Shi LZ Milasta S Carter R Finkelstein D The transcription factor Myc controls metabolic reprogramming upon T lymphocyte activation. Immunity (2011) 35:871–82. 10.1016/j.immuni.2011.09.021 22195744 PMC3248798

[28] Finlay DK Rosenzweig E Sinclair LV Feijoo-Carnero C Hukelmann JL Rolf J PDK1 regulation of mTOR and hypoxia-inducible factor 1 integrate metabolism and migration of CD8+ T cells. J Exp Med (2012) 209:2441–53. 10.1084/jem.20112607 23183047 PMC3526360

[29] Oestreich KJ Read KA Gilbertson SE Hough KP McDonald PW Krishnamoorthy V Bcl-6 directly represses the gene program of the glycolysis pathway. Nat Immunol (2014) 15:957–64. 10.1038/ni.2985 25194422 PMC4226759

[30] Lochner M Berod L Sparwasser T. Fatty acid metabolism in the regulation of T cell function. Trends Immunol (2015) 36:81–91. 10.1016/j.it.2014.12.005 25592731

[31] Vander Heiden MG Plas DR Rathmell JC Fox CJ Harris MH Thompson CB. Growth factors can influence cell growth and survival through effects on glucose metabolism. Mol Cell Biol (2001) 21:5899–912. 10.1128/MCB.21.17.5899-5912.2001 11486029 PMC87309

[32] Semenza GL Roth PH Fang HM Wang GL. Transcriptional regulation of genes encoding glycolytic enzymes by hypoxia-inducible factor 1. J Biol Chem (1994) 269:23757–63. 8089148

[33] Brandstatter O Schanz O Vorac J Konig J Mori T Maruyama T Balancing intestinal and systemic inflammation through cell type-specific expression of the aryl hydrocarbon receptor repressor. Sci Rep (2016) 6:26091. 10.1038/srep26091 27184933 PMC4869119

[34] Kuwahara M Ise W Ochi M Suzuki J Kometani K Maruyama S Bach2-Batf interactions control Th2-type immune response by regulating the IL-4 amplification loop. Nat Commun (2016) 7:12596. 10.1038/ncomms12596 27581382 PMC5025763

[35] Tan TCJ Knight J Sbarrato T Dudek K Willis AE Zamoyska R. Suboptimal T-cell receptor signaling compromises protein translation, ribosome biogenesis, and proliferation of mouse CD8 T cells. Proc Natl Acad Sci U S A (2017) 114:E6117–26. 10.1073/pnas.1700939114 28696283 PMC5544288

[36] Stedman A Beck-Cormier S Le Bouteiller M Raveux A Vandormael-Pournin S Coqueran S Ribosome biogenesis dysfunction leads to p53-mediated apoptosis and goblet cell differentiation of mouse intestinal stem/progenitor cells. Cell Death Differ (2015) 22:1865–76. 10.1038/cdd.2015.57 26068591 PMC4648334

[37] Preston GC Sinclair LV Kaskar A Hukelmann JL Navarro MN Ferrero I Single cell tuning of Myc expression by antigen receptor signal strength and interleukin-2 in T lymphocytes. EMBO J (2015) 34:2008–24. 10.15252/embj.201490252 26136212 PMC4551349

[38] Liao W Lin JX Leonard WJ. Interleukin-2 at the crossroads of effector responses, tolerance, and immunotherapy. Immunity (2013) 38:13–25. 10.1016/j.immuni.2013.01.004 23352221 PMC3610532

[39] Hieronymus T Blank N Gruenke M Winkler S Haas JP Kalden JR CD 95-independent mechanisms of IL-2 deprivation-induced apoptosis in activated human lymphocytes. Cell Death Differ (2000) 7:538–47. 10.1038/sj.cdd.4400684 10822277

[40] Rongvaux A Jackson R Harman CC Li T West AP De Zoete MR Apoptotic caspases prevent the induction of type I interferons by mitochondrial DNA. Cell (2014) 159:1563–77. 10.1016/j.cell.2014.11.037 25525875 PMC4272443

[41] Morley SJ Coldwell MJ Clemens MJ. Initiation factor modifications in the preapoptotic phase. Cell Death Differ (2005) 12:571–84. 10.1038/sj.cdd.4401591 15900314

[42] Sica A Mantovani A. Macrophage plasticity and polarization: in vivo veritas. J Clin Invest (2012) 122:787–95. 10.1172/JCI59643 22378047 PMC3287223

[43] Hodge S Hodge G Flower R Reynolds PN Scicchitano R Holmes M. Up-regulation of production of TGF-beta and IL-4 and down-regulation of IL-6 by apoptotic human bronchial epithelial cells. Immunol Cell Biol (2002) 80:537–43. 10.1046/j.1440-1711.2002.01120.x 12406387

[44] Ledru E Fevrier M Lecoeur H Garcia S Boullier S Gougeon ML. A nonsecreted variant of interleukin-4 is associated with apoptosis: implication for the T helper-2 polarization in HIV infection. Blood (2003) 101:3102–5. 10.1182/blood-2002-08-2499 12515722

[45] Veenstra H Baumann R Lukey PT Beyers N Van Helden PD Walzl G. High levels of intracellular IL-4 are expressed in circulating apoptotic T cells in patients with tuberculosis and in community controls. Tuberculosis (Edinb) (2008) 88:21–30. 10.1016/j.tube.2007.09.001 17977794

[46] Henault J Riggs JM Karnell JL Liarski VM Li J Shirinian L Self-reactive IgE exacerbates interferon responses associated with autoimmunity. Nat Immunol (2016) 17:196–203. 10.1038/ni.3326 26692173 PMC4718782

[47] He J Zhang X Wei Y Sun X Chen Y Deng J Low-dose interleukin-2 treatment selectively modulates CD4(+) T cell subsets in patients with systemic lupus erythematosus. Nat Med (2016) 22:991–3. 10.1038/nm.4148 27500725

