# Supplementary material for: Cord Blood CD8+ T Cells Have a Natural Propensity to Express IL-4 in a Fatty Acid Metabolism and Caspase Activation-Dependent Manner
Source: Front Immunol. 2018 Apr 25;9:879. doi: 10.3389/fimmu.2018.00879 (PMC5996926; doi:10.3389/fimmu.2018.00879)
Supplement: Supplementary file 3 [file data_sheet_3.PDF]

Supplemental Fig. 1. Transcriptional profiling of 'T_C2' cells by RNAseq. Multi-dimensional scaling reveals a clear separation between 'T_C2' cells and naïve $CD8^+$ T cells in the first principal component

Multidimensional scaling (MDS) analysis of the normalized, filtered RNA-seq data. As in a principal component plot, the axes represent the major sources of variation in the data based on the top 500 genes with the largest standard deviations between samples; dimension 1 represents the largest source of variation, dimension 2 represents the next largest orthogonal source of variation, etc. Dimension 1 clearly captures the difference between naïve and 'Tc2' cells.

Supplemental Fig. 2. 'Tc2' differentiation is associated with increased expression of genes for fatty acid metabolism

Expression of genes involved in fatty acid metabolism is increased after activation of naive $CD8^+$ T cells with anti-CD3/CD28 antibody in the presence of IL-6+TGF-β.

Supplemental Fig. 3. Pathways differentially enriched in 'T_C2' cells

The barcode plot ranks the moderated t-statistics of the genes in the pathway (vertical bars) from left to right, largest to smallest, and the enrichment worm shows the relative enrichment of the genes in each part of the plot. For example, the KEGG ribosome biogenesis pathway is enriched for downregulated genes in 'T_C2' cells, whereas the other pathways shown are enriched for upregulated genes.

Supplemental Tables

Supplemental Table 1: Genes differentially expressed between naïve CD8$^+$ T cells and 'T_C2' cells

The "logFC" column contains the estimate of the log_2-fold-change corresponding to the effect of interest for each gene. The "AveExpr" column represents the average log_2-expression for each gene across all samples. The "t" column contains the moderated t-statistics for each gene. The "P.Value" column contains the raw p-values for each gene, whilst the "adj.P.Val" column contains the p-values adjusted for false discovery rate using the Benjamini-Hochberg method. The "B" column represents the log-odds that each gene is differentially expressed.

Supplemental Table 2: Pathways differentially enriched between naïve CD8$^+$ T cells and 'T_C2' cells

Supplemental Table 3: Patient characteristics

Supplemental Fig.1.

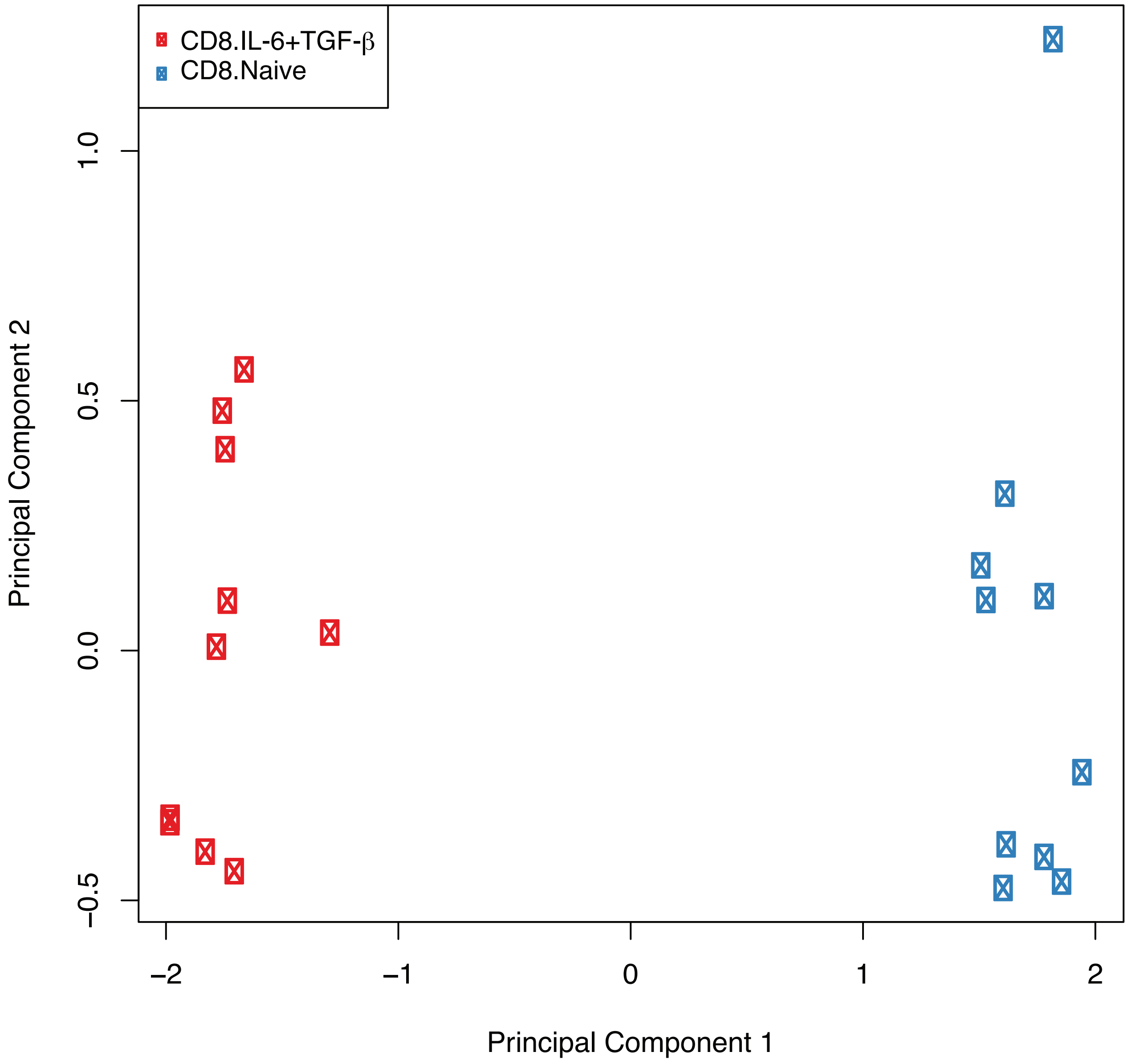

Supplemental Fig. 2

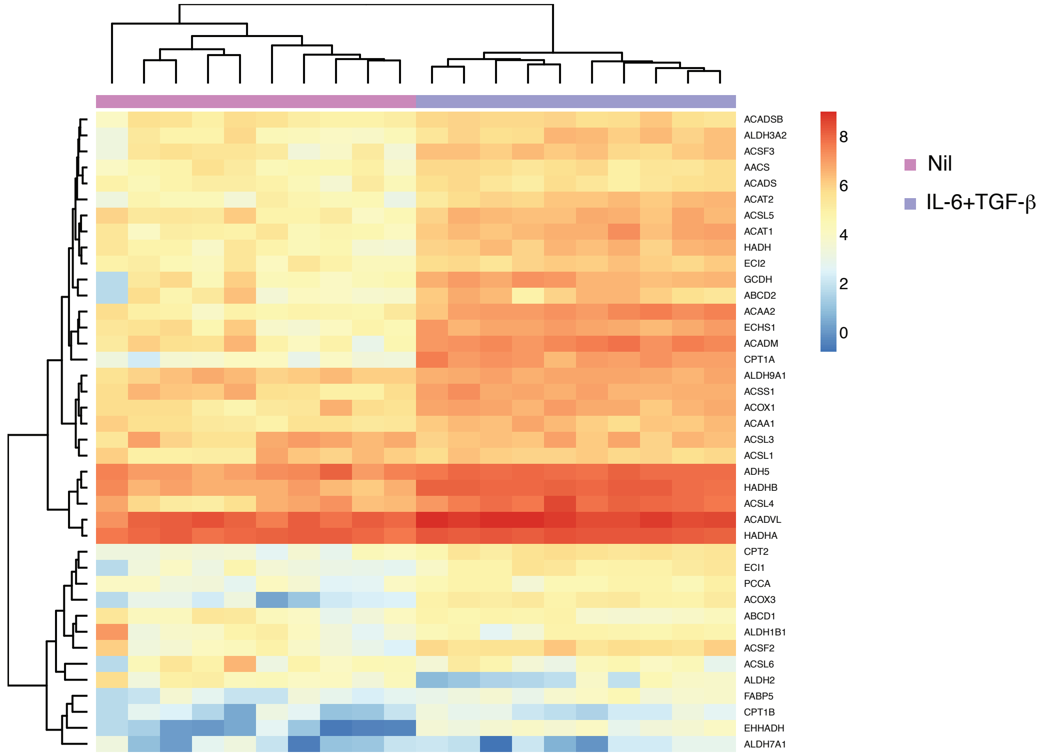

Supplemental Fig.3.

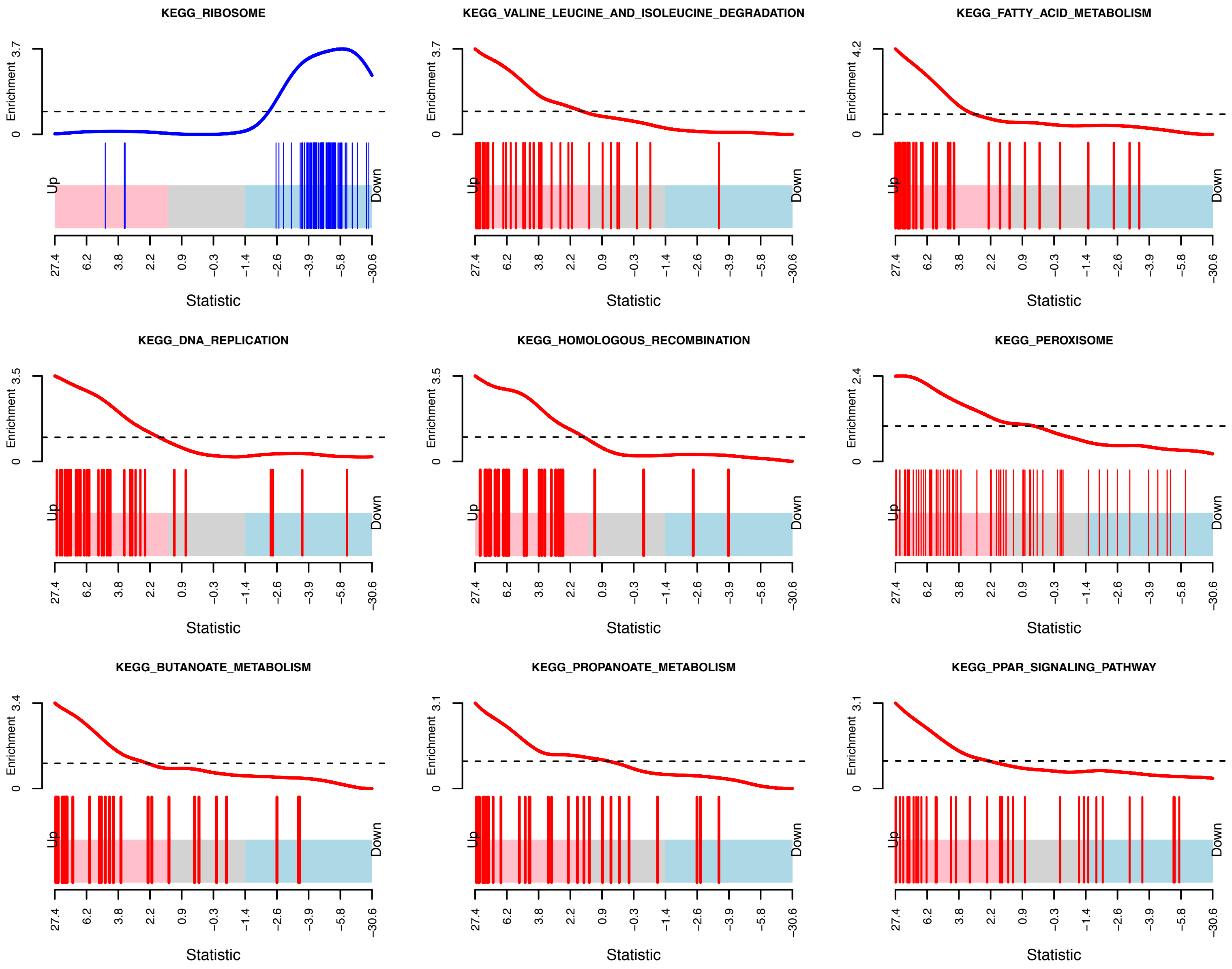

Supplemental table 3: patient characteristics

Lab Id	Gender	Age	Weight (kg)	Total-IgE	Specific-IgE	Colonoscopy appearance	Colon histology	Other gastrointestinal conditions/history	Diagnosis
FA_076	F	9	27.5	NA	Milk	Normal	Normal	Colonic polyp,<br>Colitis history	Control
FA_077	F	12	27	10	None	Normal	Normal	Gastritis	Control
FA_101	M	5	14	1100	None	Normal	Normal (Eos 17/HPF @AC)	Anal fissure	Control
FA_125	F	9	22	163	None	Normal	Normal	HP+ Gastritis	Control
FA_135	M	4	15	888	Egg, shrimp	Normal	Normal	Colonic polyp	Control
FA_143	M	8	25	31	None	Normal	Normal (Eos 15/HPF @cecum, 17/HPC @AC)	Gastritis	Control
FA_148	M	5	15	NA	None	Normal	Normal	Colonic polyp	Control
FA_155	F	7	21	11	Egg	Normal	Normal	HP+Gastritis<br>Cow's milk allergy<br>Enlarged mesenteric LNs	Control
FA_007	F	9	20.5	201	Peanut	Nodular mucosa, pale and friable， loss of vascular pattern	Large numbers of plasmablasts infiltration in lamina propria	Colonic polyp<br>JIA<br>*Toxoplasma gondii*+	Colitis
FA_051	M	1	8	128	NA	Nodular mucosa, erythema, loss of vascular pattern	Normal	Food allergy	Colitis
FA_058	M	3	16	20	Egg, Milk	Friable mucosa, with scattered follicles mostly in descending colon	Normal	Colonic polyp	Colitis
FA_061	M	7	20	17	None	Erythema throughout colon, loss of vascular pattern	Impaired epithelial integrity at cecum and transverse colon	Penicillin anaphylaxis, Gastritis	Colitis
FA_071	M	12	37	293	None	Nodular mucosa, erythema and edema	Eosinophilic colitis (Eos 24/HPF @ cecum)	Duodenal inflammation	Eosinophilic colitis
FA_078	F	7	22	103	None	Normal	Eosinophilic colitis (33/HPF ileum)	Peutz-Jeghers syndrome	Eosinophilic colitis
FA_091	F	3	13	13	Milk	Scattered follicular hyperplasia, friability, mostly in left colon	Normal	Colonic polyp	Colitis

FA_139	M	5	22	431	Egg	Pinpoint ulcers in rectal mucosa	Increased lymphocytes, plasmablasts infiltration of lamina propria	Colonic polyp history	Colitis
FA_152	M	3	13	62	NA	Normal	Eosinophilic colitis (38/HPF @cecum, 27/HPF @AC, 16/HPF @DC)		Eosinophilic colitis
FA_153	M	2	11	159	NA	Normal	Edema	Anaphylactic purpura? Bacterial colitis?	Colitis

Note:

Gender: F (female), M (male);

Specific IgE: >0.35 for egg, milk, wheat, peanut, fish, soybean, crab or shrimp;

NA: not available.

HPF: high power field,

Colon: AC: ascending colon; TC: transverse colon; DC: descending colon; SC: sigmoid colon.
